# A transcription factor DAF-5 functions in *Haemonchus contortus* development

**DOI:** 10.1186/s13071-021-05036-2

**Published:** 2021-10-12

**Authors:** Wenda Di, Fangfang Li, Li He, Chunqun Wang, Caixian Zhou, Lu Liu, Lisa Ye, Jian Chen, Min Hu

**Affiliations:** 1 State Key Laboratory of Agricultural Microbiology, Key Laboratory for the Development of Veterinary Products, Ministry of Agriculture, College of Veterinary Medicine, Huazhong Agricultural University, Wuhan, 430070 Hubei China; 2 School of Basic Medical Sciences, Hubei University of Medicine, Hubei, 442000 Shiyan China; 3 College of Animal Science and Technology, Guangxi University, Guangxi, 530004 Nanning China

**Keywords:** DAF-5, Development, Interaction, RNAi, siRNA, Transcription factor

## Abstract

**Background:**

Abnormal dauer formation gene (*daf-5*), located downstream of the DAF-7 signalling pathway, mainly functions in dauer formation and reproductive processes in the free-living nematode *Caenorhabditis elegans*. Although the structure and function of *daf-5* have been clarified in *C. elegans*, they still remain totally unknown in *Haemonchus contortus*, a socio-economically important parasitic nematode of gastric ruminants.

**Methods:**

A homologue of *daf-5*, *Hc-daf-5*, and its inferred product (*Hc*-DAF-5) in *H. contortus* were identified and characterized in this study. Then the transcriptional profiles of *Hc-daf-5* and the anatomical expression of *Hc*-DAF-5 in *H. contortus* were studied using an integrated molecular approach. RNA interference (RNAi) was performed to explore its function in transition from the exsheathed third-stage larvae (xL3s) to the fourth-stage larvae (L4s) in vitro. Finally, the interaction between *Hc*-DAF-5 and *Hc*-DAF-3 (a co-Smad) was detected by bimolecular fluorescence complementation (BiFc) in vitro.

**Results:**

It was shown that *Hc-*DAF-5 was a member of the Sno/Ski superfamily. *Hc-daf-5* was transcribed in all developmental stages of *H. contortus*, with significant upregulation in L3s. Native *Hc-*DAF-5 was localized in the reproductive organs, cuticle, and intestine via immunohistochemistry. RNAi revealed that specific small interfering RNAs (siRNAs) could retard xL3 development. In addition, the interaction between *Hc*-DAF-5 and *Hc*-DAF-3 indicated that the SDS box of *Hc*-DAF-5 was dispensable for the binding of *Hc*-DAF-5 to *Hc*-DAF-3, and the MH2 domain was the binding region between *Hc*-DAF-3 and *Hc*-DAF-5.

**Conclusions:**

In summary, these findings show that *Hc-daf-5* functions in the developmental processes of *H. contortus*, and this study is the first attempt to characterize the *daf-5* gene in parasitic nematodes.

**Graphical abstract:**

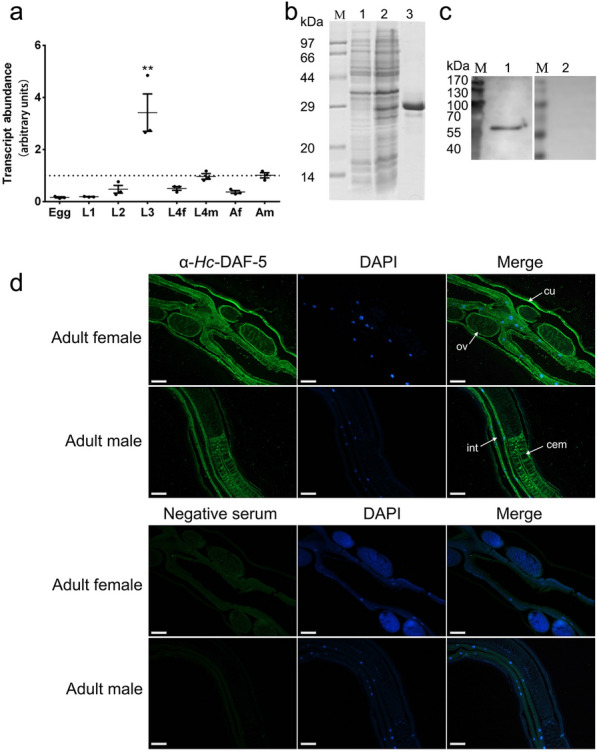

**Supplementary Information:**

The online version contains supplementary material available at 10.1186/s13071-021-05036-2.

## Background

Transcription factors function in basal transcription by binding to particular DNA sequences in gene regulatory regions to control their transcription. Transcription factors are commonly classified into activator and repressor families based on their functions. Although the majority of transcription factors have a positive effect, a number of transcription factors exert an inhibitory effect on transcription [1].

For the transforming growth factor-β (TGF-β) signalling pathway in vertebrates, activation of receptors causes downstream R-Smad phosphorylation, which allows the latter to form an active Smad transcriptional complex with co-Smads. The function of this complex is affected positively or negatively by interaction with co-factor, co-activator, and co-repressor molecules, which ultimately regulates cellular processes such as proliferation, differentiation, and apoptosis [2]. As downstream components of the TGF-β signalling pathway, the Ski proto-oncoprotein family of co-repressors, including c-Ski and SnoN, play important roles in tightly controlled repression of Smad-mediated transcriptional activation, and antagonize TGF-β signalling by binding to the Smad transcriptional complex, thus preventing their interaction with co-activators, and recruiting co-repressors [3]. Additionally, Sno/Ski transcription is regulated by Smads. Thus, the degradation or accumulation of Sno/Ski protein occurs depending on the context of the TGF-β signalling [4].

DAF-5, the homologue of vertebrate Ski/Sno in the free-living nematode *Caenorhabditis elegans*, functions in the TGF-β signalling pathway (DAF-7 signalling pathway) for dauer regulation and egg-laying in this nematode, but not like a conventional Sno/Ski protein [5]. In brief, the DAF-7 signal is transduced by R-Smads. These molecules, when activated, function as a transcriptional complex and inhibit the functions of DAF-5. DAF-5 acts as a co-factor of co-Smad (DAF-3), rather than an antagonist of DAF-3. It also acts as a complex and is antagonized by upstream R-Smad components [5].

Although the functions of Sno/Ski and DAF-5 have been clearly identified in vertebrates and *C. elegans*, respectively, their roles in parasitic nematodes have not yet been elaborated. Elucidating the functions of DAF-5 in parasitic nematodes will help us understand how this molecule and the DAF-7 signalling pathway work in the parasites. Herein, a homologue of *daf-5* was identified from *Haemonchus contortus*, a blood-sucking gastric parasitic nematode of small ruminants. Its temporal transcriptional profiles in different developmental stages and special expression patterns in adult worms were explored by real-time polymerase chain reaction (PCR) and immunohistochemistry, respectively. In addition, small interfering RNAs (siRNAs) were used to knock down *Hc-daf-5* in exsheathed third-stage larvae (xL3s) by soaking. A bimolecular fluorescence complementation (BiFc) experiment was also performed to demonstrate the interaction between *Hc*-DAF-5 and *Hc*-DAF-3.

## Methods

### Worm maintenance

The *H. contortus* Haecon-5 strain was maintained by goats, which were infected orally with 5000 L3s. Free-living stages including eggs, first-stage larvae (L1s), second-stage larvae (L2s), and L3s were harvested from faeces of infected goats. Parasitic stages (L4s and adults) were collected from the abomasa of infected goats euthanized at 8 and 30 days, respectively, and these two developmental stages were washed extensively in 0.85% sodium chloride, and male and female worms were carefully separated under a microscope prior to storage at −80 °C.

### Nucleic acid and protein preparation and gene cloning

Total RNA was extracted from individual developmental stages of *H. contortus* using TRIzol reagent (Simgen, China). All RNA samples were treated with DNase I to remove genomic DNA (gDNA), and then their integrity and yields were verified by electrophoresis and spectrophotometric analysis. Complementary DNA (cDNA) was synthesized from RNA (1 μg) using the PrimeScript RT reagent kit with gDNA Eraser (Takara, Japan) for coding sequence (CDS) amplification and real-time PCR. RNA was stored at −80 °C and DNA was stored at −20 °C until use.

The whole-worm proteins were prepared by grinding adult worms in phosphatase inhibitor and protein lysate (Roche Molecular Biochemicals, Switzerland), which were fractionated into soluble and insoluble materials after centrifugation at 10,000×*g* for 3 min at 4 °C. Then the soluble material was stored at −80 °C with protease inhibitor (Thermo Fisher, USA) added, followed by western blot analysis.

The sequences of full-length cDNA and gDNA, were retrieved from the transcriptomic and genomic datasets of *H. contortus*. The CDS was amplified from cDNA with the primer pair Hc-daf-5-cF/Hc-daf-5-cR (Additional file 1: Table S1) using the following protocol: 98 °C/10 min, then 98 °C/10 s, 55 °C/5 s and 72 °C/2 min for 35 cycles, and 72 °C/10 min. The PCR product was inserted into pMD-19T and sequenced in both directions directly by Tsingke Biological Technology, China.

### Bioinformatics analyses

The CDS of *Hc-daf-5* were conceptually translated into predicted amino acids using DNASTAR software (http://www.dnastar.com/). The predicted amino acid sequence of *Hc*-DAF-5 was compared with the sequences in non-redundant protein databases using BLASTP from the National Center for Biotechnology Information (NCBI) to confirm the homologous sequences.

The structural domains (Dach and SDS boxes) of *Hc*-DAF-5 were aligned with its homologues from 11 species (*Ancylostoma ceylanicum*, *Brugia malayi*, *Caenorhabditis briggsae*, *C. elegans*, *Danio rerio*, *Drosophila melanogaster*, *Equus caballus*, *Homo sapiens*, *Mus musculus*, *Nippostrongylus brasiliensis*, and *Toxocara canis*) [6–20] (Additional file 1: Table S2) using the BioEdit program. For *Hc*-DAF-5, the coiled coil region was predicted by Expasy (https://embnet.vital-it.ch/software/COILS_form.html).

The full-length protein sequences from eight species (*C. briggsae*, *C. elegans*, *D. rerio*, *D. melanogaster*, *H. sapiens*, *M. musculus*, *N. brasiliensis*, and *T. canis*) (Additional file 1: Table S2) were aligned and used for phylogenetic analyses by means of the neighbour-joining (NJ), maximum parsimony (MP), and maximum likelihood (ML) methods. Confidence limits were assessed using a bootstrap procedure employing 1000 pseudo-replicates in MEGA [21].

### Transcriptional analyses of *Hc-daf-5* in different stages of *H. contortus* by real-time PCR

Real-time PCR was carried out using the specific primers Hc-daf-5-qF/Hc-daf-5-qR (Additional file 1: Table S1) to determine the mRNA levels in different developmental stages of *H. contortus* including eggs, L1s, L2s, L3s, both sexes of L4s, and adult worms. Total RNA of each stage was isolated with TRIzol reagent according to the manufacturer’s instructions and treated with DNase I to remove gDNA before synthesis of cDNA. The real-time PCR conditions were set as follows: one cycle at 50 °C/2 min and 95 °C/30 s, 40 cycles at 95 °C/15 s, 60 °C/15 s, and 72 °C/20 s, and one cycle at 60 °C/1 min, 95 °C/15 s, and 60 °C/15 s. Each sample was tested in triplicate, with β-tubulin of *H. contortus* (GenBank: M76493) as a reference gene (using specific primers Hctubulin-qF and Hctubulin-qR; Additional file 1: Table S1), and the average threshold (Ct) was taken to compare the relative quantities with Am (Am = 1) using the 2^−△△Ct^ method [22]. This assay was carried out three times. One-way analysis of variance (ANOVA) in GraphPad Prism 6 was adopted for statistical analysis. *P*-values were calculated using the Tukey post hoc test, and *P* < 0.05 was considered statistically significant.

### Production of polyclonal antibody against recombinant *Hc*-DAF-5 and immunoblot analysis

A pair of specific primers Hc-daf-5-pF/Hc-daf-5-pR (Additional file 1: Table S1) containing double restriction sites was designed according to the CDS of *Hc-daf-5*, and it was employed to amplify the CDS of *Hc-daf-5* through PCR. Then the amplified sequence was cloned into the prokaryotic expression vector to create the expression plasmid pET-28a-Hc-daf-5, which was transformed into BL21 (DE3) cells of *Escherichia coli*, followed by 1 mM isopropyl β-d-1-thiogalactopyranoside (IPTG) induction at 16 °C for 12 h to produce recombinant r*Hc*-DAF-5. Next, the recombinant r*Hc*-DAF-5 was purified using a Ni Sepharose column system (GE, USA) according to the manufacturer’s protocol. Later, the purified r*Hc*-DAF-5 was used in immunizing New Zealand white rabbit to produce anti-*Hc*-DAF-5 polyclonal antibody. Finally, the titer and specificity of anti-*Hc*-DAF-5 polyclonal antibody were determined by enzyme-linked immunosorbent assay (ELISA) and western blot.

Western blot was performed as follows. Proteins were resolved by 12% sodium dodecyl sulfate–polyacrylamide gel electrophoresis (SDS-PAGE) and transferred onto an Immobilon^®^-PSQ membrane (Merck Millipore Ltd.). Then the immunoblot membrane was blocked with blocking buffer [1% (w/v) bovine serum albumin (BSA) (BioFroxx, China)] in phosphate-buffered saline with 20% Tween-20 (PBST)] for 6 h at 4 °C, washed three times with PBST, and incubated with *Hc*-DAF-5 antiserum (1:1000 in PBST) overnight at 4 °C. Next, samples were washed six times in PBST and subsequently incubated with horseradish peroxidase (HRP)-conjugated goat anti-rabbit antibody (1:1000, Beyotime Biotechnology, China) for 2 h at 37 °C, followed by washing an additional five times. Finally, immunodetection was performed by chemiluminescence (WesternBright ECL kit; K-12045-D10, China), and images were acquired using the ChemiDoc XRS+ system (Bio-Rad, USA).

### Evaluation of protein expression in *H. contortus *via immunofluorescence assay

Fresh male and female adults of *H. contortus* were fixed in 4% paraformaldehyde at 4 °C for 24 h and then consecutively dehydrated in an ethanol series (75% for 4 h, 85% for 2 h, 90% for 2 h, 95% for 1 h, and 100% for 30 min twice). The dehydrated worms were then incubated in xylene/absolute ethanol (1:1) solution for 5 min and xylene for 10 min, and then embedded in paraffin. Next, 4 μm-thick paraffin-embedded sections were subjected to immunofluorescence staining. For immunofluorescence assay, the sections were treated with EDTA buffer at 100 °C for 10 min. After blocking with 5% BSA for 20 min, anti-*Hc*-DAF-5 polyclonal antibody and goat anti-rabbit IgG antibody diluted at 1:100 were sequentially added and incubated at 4 °C overnight and at 37 °C for 50 min. Then the sections were stained with 4′6-diamidino-2-phenylindole (DAPI) for 5 min at 37 °C in a dark place. After thorough washing with PBS, the sections were observed under a fluorescence microscope (Olympus CX21, Japan).

### RNA interference (RNAi) in* H. contortus* by soaking in siRNA

The CDS of *Hc-daf-5* was used to design the siRNA sequences with the siRNA Design Tools program, and siRNA oligos (Additional file 1: Table S3) were chemically synthesized by Shanghai GenePharma Co., Ltd.

For RNAi, L3s were exsheathed and washed five times in 0.9% NaCl solution, followed by centrifugation at 600×*g* with diethyl pyrocarbonate (DEPC)-treated water three times. In each silencing assay, 50 μl of nematode suspension (about 5000 larvae) was placed into a 96-well plate. Then 10 μl of Lipofectamine 2000 (Thermo Fisher, USA) was incubated with 5 μl of Earle's Balanced Salt Solution (EBSS) (pH 5.2) containing 2.5 μg/μl amphotericin, 100 μg/μl streptomycin, and 100 IU/ml penicillin (Gibco, USA) at 25 °C for 5 min. RNasin (0.2 U) and siRNA solutions were added for incubation for 15 min. Three siRNAs of *Hc-daf-5* were mixed in equal amounts, and the final concentration of each siRNA was adjusted to 1 μM, while the final concentration of negative control siRNA was 3 μM, with nuclease-free water as the blank control.

The knockdown experiments were carried out as described previously [23]. In brief, three groups of xL3s (5000 in each group), which were kept in 80 μl of EBSS (pH 5.2) and supplemented with respective siRNAs, were soaked for 72 h. Approximately 100 larvae in each group were transferred to a fresh culture medium with EBSS for another 5 days to assess their development. The remaining larvae were subjected to RNA extraction.

All RNAs were extracted using TRIzol reagent according to the manufacturer’s instructions. Then cDNA was synthesized using the PrimeScript RT reagent kit with gDNA Eraser (Takara, China). Each 10 μl of reaction was conducted with the same amount of cDNA from each sample. The *18S* gene of *H. contortus* was taken as the endogenous control using the primer pair Hc-18S-qF/Hc-18S-qR (Additional file 1: Table S1). Primers (Hc-daf-5-qF/Hc-daf-5-qR) used in detecting transcriptional profiles were also used here for the detection of transcriptional changes of *Hc-daf-5* in worms treated with siRNA. The amplification efficiency of the primers was tested by a standard curve assay, and linear regression analysis showed similar slopes of all tested primers. The real-time PCR was performed on an ABI 7100 thermal cycler (Applied Biosystems, Germany) as per the following conditions: one cycle at 50 °C/2 min and 95 °C/30 s, 40 cycles at 95 °C/15 s, 60 °C/15 s, and 72 °C/20 s, and a cycle at 60 °C/1 min, 95 °C/15 s, and 60 °C/15 s. The fold change of *Hc-daf-5* expression after RNAi was calculated by the 2^−ΔΔCt^ method. ΔΔCt = [(^Ct^ RNAi, *Hc-daf-5*)  − (^Ct^ RNAi, *Hc-18S*)] − [(^Ct^ Blank, *Hc-daf-5*) − (^Ct^ Blank, *Hc-18S*)] [24].

### Assessing the interaction between *Hc*-DAF-5 and *Hc*-DAF-3 by bimolecular fluorescence complementation (BiFC)

The BHK21 cells were grown and maintained in Dulbecco's modified Eagle medium (DMEM) supplemented with 10% fetal bovine serum (FBS) and 1% penicillin/streptomycin. The plasmids pbJun-HA-KN151 and pbFos-Myc-LC151 were used as original plasmids for plasmid construction. In brief, the CDS fragment of *Hc-daf-3* was amplified with the primer pair Hc-daf-3-fF/Hc-daf-3-fR (Additional file 1: Table S1) and cloned into pbJun-HA-KN151 between the NheI and XhoI sites to replace *bJun*, thus generating the p*Hc*DAF3-HA-KN151 plasmid. Meanwhile, the CDS fragment of *Hc-daf-5* was amplified with the primer pair Hc-daf-5-fF/Hc-daf-5-fR (Additional file 1: Table S1) and cloned into pbFos-Myc-LC151 between the NheI and PvuI sites to replace *bFos*, so as to generate the p*Hc*DAF5-Myc-LC151 plasmid. The BHK21 cells were seeded onto six-well plates and grown to 70–80% confluence for transfection. To examine the interactions between *Hc*-DAF-3 and *Hc*-DAF-5, the plasmids p*Hc*DAF3-HA-KN151 and p*Hc*DAF5-Myc-LC151 were co-transfected using Lipofectamine 2000 (Thermo Fisher, USA). Then the cells were incubated at 37 °C with 5% CO_2_ for a further 20 h, and fluorescence was subsequently detected at 580–680 nm and imaged.

In addition to the full-length constructs of *Hc*-DAF-3, a DNA fragment encoding the *Hc*-DAF-3-MH2 domain (645 bp; 484–697 amino acids [aa]) was amplified with the primer pair Hc-daf-3-MH2-F/Hc-daf-3-fR (Additional file 1: Table S1), sequenced, and cloned into p*Hc*DAF3-MH2-HA-KN151. Similarly, a DNA fragment encoding the *Hc*-DAF-5-SDS domain (900 bp; 1–300 aa) was amplified with the primer pair Hc-daf-5-fF/Hc-daf-5-SDS-R (Additional file 1: Table S1), sequenced, and cloned into p*Hc*DAF5-SDS-Myc-LC151. Finally, the plasmids pbFos-Myc-LC151 and pbJun-HA-KN151, which express bJun and bFos, respectively, were co-transfected as positive controls, while pHA-KN151 and p*Hc*DAF5-SDS-Myc-LC151 were set as negative controls.

## Results

### Sequence and phylogenetic analyses of *Hc*-DAF-5

The CDS of *Hc-daf-5* was 1629 bp in length (GenBank accession number: MK159305), and it was predicted to encode a protein (*Hc*-DAF-5) of 542 amino acids. The full-length genomic sequence of *Hc-daf-5* was 8535 bp long, containing 11 exons (73–207 bp) and 10 introns (57–3071 bp). Compared with *Ce-daf-5* [5], *Hc-daf-5* had more exons and introns.

A BLAST search with the *Hc*-DAF-5 protein sequences as bait against the NCBI GenBank databases revealed similarities to the SKI, SnoN, Skate, and Icy in *H. sapiens* and the Snowski and Iceskate in *D. melanogaster* (Additional file 1: Table S2) [25, 26]. The predicted protein sequence of *Hc*-DAF-5 was then aligned with homologues from five nematodes and five other metazoans. Multiple sequence comparison revealed the highest identity of *Hc*-DAF-5 to SKI in *N. brasiliensis* (58.3%), but low identity ranging from 18.7 to 28.7% to its homologues from different metazoan species, including *B. malayi* (Snowski, 22.7%), *C. briggsae* (DAF-5, 21.8%), *C. elegans* (DAF-5, 23.0%), *D. rerio* (SKI, 24.1%), *D. melanogaster* (Iceskate, 20.3%; Snowski, 20.9%), *E. caballus* (SKI, 22.9%), *H. sapiens* (Icy, 18.7%; Skate, 21.1%; SKI, 23.1%; SnoN, 20.4%), *M. musculus* (SKI, 21.9%), and *T. canis* (SKI, 28.7%).

**Fig. 1:**
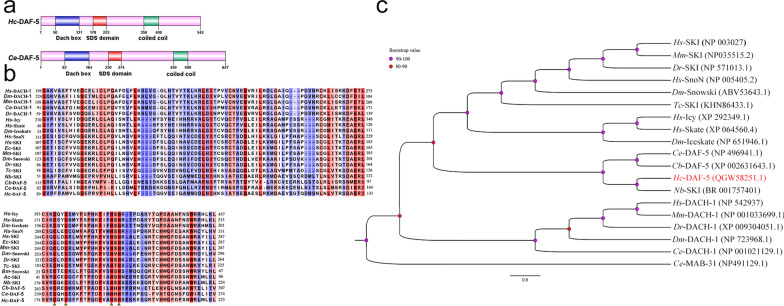
*Hc*-DAF-5 displays conserved features with homologues of Sno/Ski proteins. **a** Primary structures of *Hc*-DAF-5 and *Ce*-DAF-5 with signature domains and motifs. **b** Amino acid sequence alignment of Dach boxes (above) and SDS (below) of Sno/Ski proteins from *Haemonchus contortus* and other species. The triangle symbols below the SDS box represent conserved zinc-chelating residues. Accession numbers and abbreviations: *Hs*, *H. sapiens* (DACH1-NP_723968.1); *Dm*, *D. melanogaster* (DACH1-NP_723968.1); *Mm*, *M. musculus* (DACH-1-NP_001033699.1); *Ce*, *C. elegans* (DACH-1-NP_001021129.1); *Dr*, *D. rerio* (DACH1-XP_009304051.1); *Hs*, (Icy-XP_292349; Skate-XP_064560); *Dm,* (Iceskate-NP_651946.1); *Hs*, (SnoN-NP_005405.2; SKI-NP_003027); *Dm,* (Snowski-ABV53643.1;); *Ec*, *E. caballus* (SKI-NP_001075287.1); *Mm*, (SKI-NP035515.2); *Dr*, (SKI-NP_571013.1); *Tc*, *T. canis* (SKI-KHN86433.1); *Bm*, *B. malayi* (Snowski-AAQ93809.1); *Ac*, *A. ceylanicum* (SKI-EYB97253.1); *Nb*, *N. brasiliensis* (SKI-found in WormBase as NBR_0001757401); *Cb*, *C. briggsae* (DAF-5-XP_002631643.1); *Ce*, *C. elegans* (DAF-5-NP_496941.1). **c** A rooted neighbour-joining tree showing the relationships of DAF-5 in *H. contortus* with 17 homologues from eight other organisms. The tree is calculated using the Jones-Taylor-Thornton model in MEGA 6.0. Nodal support values for each clade are colour-coded. These eight species include four nematodes (*C. briggsae*: DAF-5; *C. elegans*: DAF-5, DACH-1, MAB-31; *N. brasiliensis*: SKI; *T. canis*: SKI), one insect (*D. melanogaster*: Snowski, Iceskate, Dachshund), one fish (*D. rerio*: SKI, Dachshund), and two mammals (*H. sapiens*: SKI, SnoN, Icy, Skate, DACH-1; *M. musculus*: SKI, Dachshund). The accession numbers of 17 molecules are given to the right of each molecule’s name. *Ce*-MAB-31 is used as the outgroup

The alignment of these sequences also indicated that *Hc*-DAF-5 had two functional domains including a Dach box (Dachshund-homology domain) and an SDS box (Smad binding domain) (Fig. 1a). Dach box is a domain shared by the Sno/Ski, Skate/Icy and Dachshund superfamily. Dachshund is a transcription regulator conserved throughout bilaterians. Alignment of the Dachshund-homology domain among selected species (Fig. 1b, above) revealed that Dach box of *Hc*-DAF-5 had higher similarity to that of *Nb*-SKI than to that of *Ce*-DAF-5 and *Cb*-DAF-5. In *H. contortus*, DAF-5 clearly possessed an SDS box in the Smad binding domain, which was not found in any other proteins in *H. contortus* (Fig. 1a). The SDS box of *Hc*-DAF-5 had higher similarity to that of the Snowski group than to that of the Iceskate group. In human SKI, the SDS box and about 20 amino acids on either side constituted the minimal region required for Smad4 binding, so this region was also used for alignment (Fig. 1b below). All members of this family had a conserved zinc finger in the SDS box. The SDS box of *Hc*-DAF-5 had higher similarity to that of parasitic nematodes (such as *B. malayi*) than to that of *C. elegans* (Fig. 1b, below). As with *Ce*-DAF-5, *Hc*-DAF-5 also had a predicted coiled coil structure at the C-terminus (Fig. 1a).

The results of phylogenetic analysis showed that the topologies of the MP, ML, and NJ trees were concordant. *Hc*-DAF-5 grouped with *Nb*-SKI with 100% bootstrap support, and then they grouped with DAF-5 from *C. elegans* and *C. briggsae* with 92% bootstrap support. This DAF-5 cluster grouped with Ski/Sno and Ice/Skate from *H. sapiens*, *M. musculus*, *D. rerio*, *D. melanogaster*, and *T. canis*, with 85% bootstrap support. The Dachshund subfamily from *C. elegans*, *D. melanogaster*, *D. rerio*, *H. sapiens*, and *M. musculus* constituted an independent cluster with 99% bootstrap support (Fig. 1c).

### Transcription of *Hc-daf-5 *and expression of *Hc*-DAF-5 in *H. contortus*

**Fig. 2:**
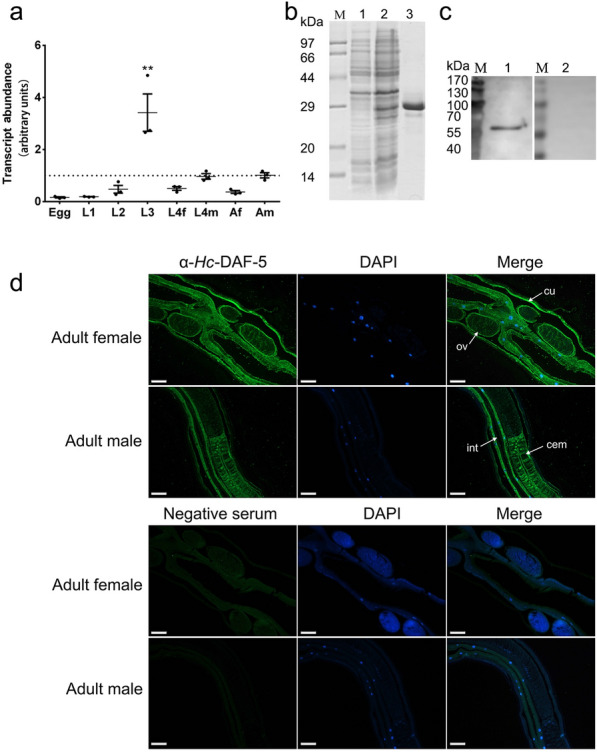
Spatiotemporal expression of *Hc*-DAF-5 in *H. contortus*. **a** Transcriptional levels of *Hc-daf-5* in different developmental stages of *H. contortus*. Transcript abundance is detected and compared among eight developmental stages including eggs (Egg), the first-stage larvae (L1), the second-stage larvae (L2), the third-stage larvae (L3), the fourth-stage female (L4f), the fourth-stage male (L4m), adult female (Af), and adult male (Am) of *H. contortus*. The relative quantities (compared with Am, Am = 1, the dotted horizontal line) are shown. All gene transcription levels are normalized to that of the β-tubulin gene. Data are presented as mean ± SEM from three independent experiments. Significant difference is indicated by ** (*P* < 0.01). **b** SDS-PAGE analyses of recombinant *Hc*-DAF-5. Lane M: standard protein molecular weight marker. Expression of *Hc*-DAF-5 is induced with 1 mM IPTG. Lane 1: recombinant *Hc*-DAF-5 expressed in *E*. *coli* before induction. Lane 2: recombinant *Hc*-DAF-5 expressed after induction. Lane 3: purified recombinant *Hc*-DAF-5. **c** Western blot analyses using total *H. contortus* protein. Lane 1: detection of *Hc*-DAF-5 by the rabbit antiserum containing anti-r*Hc*-DAF-5 antibody. Lane 2: the same blot re-probed with pro-immune serum (as control). **d** Localization of *Hc*-DAF-5 in adult worms. Images in line 1 and line 2 are taken under UV light corresponding to green fluorescent protein (GFP) and DAPI, and merged images are given in line 3. Scale bars: 100 μm. cu (cuticle), ov (ovary), int (intestine), cem (cement gland)

The transcriptional profile of *Hc-daf-5* was explored in eight developmental stages and sexes including egg, L1, L2, L3, L4 female, L4 male, adult female, and adult male throughout the life cycle of *H. contortus*. *Hc-daf-5* was transcribed at a detectable level in all tested stages and sexes, with significant upregulation in L3 (ANOVA, *F*_(7, 16)_ = 12.03, L3 vs egg, L1, L2, L4f, L4m, and Af, *P* ≤ 0.0001. L3 vs Am, *P* = 0.001) (Fig. 2a).

Truncated *Hc*-DAF-5 (108–297 aa) was expressed in *E. coil* BL21 (DE3) with a size of 27.5 kDa (Fig. 2b). The anti-r*Hc*-DAF-5 polyclonal antibody was used in western blot, and a band of around 60 kDa in total protein extracted from adult worms was recognized, while no band was recognized by the negative serum (Fig. 2c). In further immunofluorescence assay, the anti-r*Hc*-DAF-5 polyclonal antibody detected the endogenous *Hc*-DAF-5 expression in the cuticle and intestine of both female and male adult worms, as well as in the ovary of female adult worms and the cement gland of male adult worms. No fluorescence was observed in worm sections probed with the negative serum (Fig. 2d).

### Effect of specific siRNA on xL3 development of *H. contortus*

**Fig. 3:**
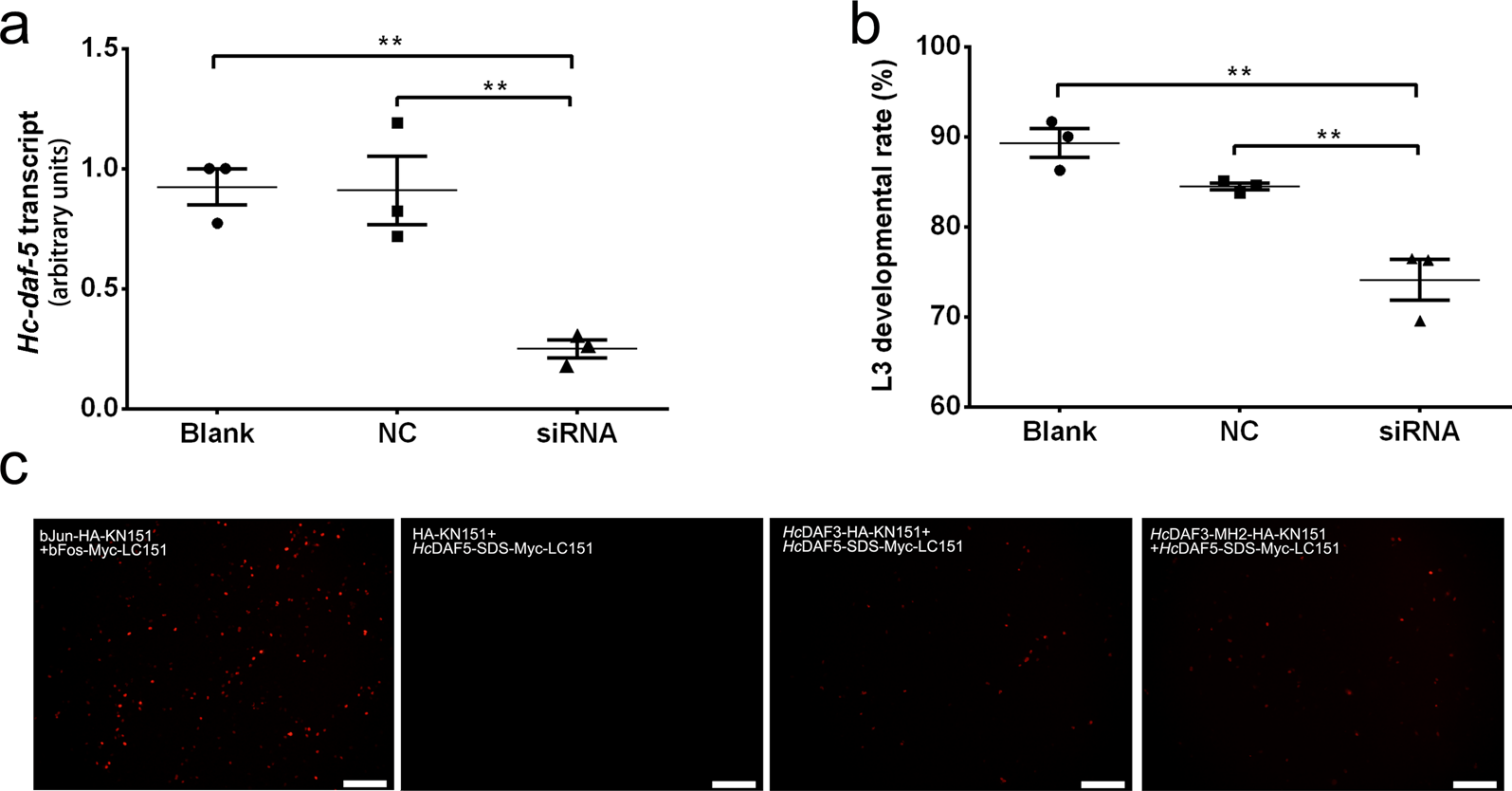
*Hc*-DAF-5 is involved in differentiation from xL3 to L4 and interacts with *Hc*-DAF-3 in *H. contortus*. **a**, **b** The effects of RNAi on the development of *H. contortus* xL3 in vitro. **a** The transcriptional changes of *Hc-daf-5* in *H. contortus* after soaking for 72 h, detected by real-time PCR. **b** L3 developmental rate in vitro for 5 days following RNAi. *P < 0.05; **P < 0.01; ***P < 0.001. Graphs in panels **a** and **b** show mean ± SEM from three independent experiments. **c** Assessment of the interaction between *Hc*-DAF-3 and *Hc*-DAF-5 in live mammalian cells using BiFC. BHK21 cells are co-transfected with plasmids, and images are acquired at 20 h after transfection. Scale bars: 100 μm. Full-length *Hc*-DAF-3 interacts with the SDS box of *Hc*-DAF-5 (*Hc*DAF3-HA-KN151 and *Hc*DAF5-SDS-Myc-LC151), while the MH2 domain of *Hc*-DAF-3 also interacts with the SDS box of *Hc*-DAF-5 (*Hc*DAF3-MH2-HA-KN151 and *Hc*DAF-5-SDS-Myc-LC151). Positive controls (bJun-HA-KN151 and bFos-Myc-LC151) and negative controls (HA-KN151 and *Hc*DAF5-SDSMyc-LC151) are also shown

As observed in previous studies, the development of *H. contortus* from xL3 to L4 underwent six stages of morphological changes, namely stage A to stage F [27, 28]. In RNAi assay herein, the soaking of xL3s in *Hc-daf-5*-specific siRNA for 72 h resulted in a significant reduction in *Hc-daf-5* transcript abundance, whereas no reduction in *Hc-daf-5* transcript abundance was detected in the control siRNA-treated group or untreated group (ANOVA, *F*_(2, 6)_ = 16.57, *Hc-daf-5* siRNA-treated group versus control siRNA group, versus blank group, both *P* = 0.0060) (Fig. 3a). In addition, the proportion of xL3s developed to stage B and beyond in the *Hc-daf-5* siRNA-treated group was significantly lower than that in the control siRNA-treated group and untreated group (ANOVA, (*F*_(2, 6)_ = 23.65, *Hc-daf-5* siRNA-treated group versus control siRNA group, versus blank group, *P* = 0.0054 and *P* = 0.0015, respectively) (Fig. 3b).

### *Hc*-DAF-5 interacts with co-Smad *Hc*-DAF-3

BiFC was employed to detect the interaction between *Hc*-DAF-5 and *Hc*-DAF-3 in live mammalian cells. The cells co-transfected with plasmid constructs as the positive controls encoding bJun-HA-KN151 and bFos-Myc-LC151 showed bright red fluorescence, consistent with the result in a previous report (Fig. 3c) [29]. No red fluorescence was observed in cells harbouring HA-KN151 and SDS of *Hc*-DAF-5 (*Hc*DAF5-SDS-Myc-LC151) protein pair, or in the blank control (not shown). In two experimental groups, red fluorescence was observed in cells harbouring full-length *Hc*-DAF-3 (*Hc*DAF3-HA-KN151) and SDS of *Hc*-DAF-5 as well as in cells harbouring MH2 of *Hc*-DAF-3 (*Hc*DAF3-MH2-HA-KN151) and SDS of *Hc*-DAF-5. However, the cells harbouring full-length *Hc*-DAF-3 and full-length *Hc*-DAF-5 (*Hc*DAF5-Myc-LC151) protein pair, or MH2 of *Hc*-DAF-3 and full-length *Hc*-DAF-5 protein pair displayed no red fluorescence (not shown).

## Discussion

In the present study, *Hc-daf-5*, a homologue of *Ce-daf-5* and a transcription factor-encoding gene, was identified and characterized in *H. contortus*. Sequence analysis revealed that the predicted protein *Hc*-DAF-5 contained a Dach box, an SDS box, and a coiled coil motif. Approximately 2–3% of all protein residues form a coiled coil motif, and it functions by mediating oligomerization of a large number of protein subunits. It can participate in signal-transducing events or act as a molecular recognition system [30]. The conservation of the coiled coil motif between *Hc*-DAF-5 and *Ce*-DAF-5 indicates the potential function of *Hc*-DAF-5 in transcriptional regulation [30].

Recent research has shown that the insulin-like signalling pathway and dafachronic acid (DA) signalling pathway play a conserved role in larval development in both free-living and parasitic species [31–34]. In contrast, the role of the TGF-β signalling pathway does not seem to be conserved between free-living and parasitic species in larval development regulation [35]. In *C. elegans*, *Ce-daf-5* is transcribed in all developmental stages including dauer larvae. Its level peaks in L1s and decreases thereafter (https://wormbase.org). However, in *Strongyloides stercoralis*, the transcription of *Ss-daf-5* is higher in infective third-stage larvae (L3i) [36]. In the present study, the transcription of *Hc-daf-5* remained low in L1s and L2s, but peaked in L3i, which is different from that of *Ce-daf-5* but similar to that of *Ss-daf-5*. In addition, the transcription pattern of *Hc-daf-5* is consistent with that of genes of the DAF-7 signalling pathway in *H. contortus*, including *Hc-daf-3* and *Hc-tgfbr-2* [37, 38]. The difference in transcriptional profiles of *daf-5* between *C. elegans* and parasitic nematodes (including *H. contortus* in clade V and *S. stercoralis* in clade IV herein), together with that in previous studies [38], indicates divergent functions of the TGF-β signalling pathway in free-living and parasitic species, suggesting that parasitic nematodes utilize the existing signalling pathway for different purposes in evolution.

Considering the high transcription level of *Hc-daf-5* in L3s, siRNA-mediated RNAi was performed to explore the role of *Hc-daf-5* in transition from the free-living stage (xL3) to the parasitic stage (L4). In this study, silencing of *Hc-daf-5* decreased the developmental rate of xL3. The experimental group treated with specific siRNAs showed fewer developed worms because the buccal development failed to initiate. This is the same effect as described for the knockdown of *Hc-daf-3* and *Hc-akt-1* [38, 39], and suggests that *Hc-daf-5* functions in the development from xL3 to L4.

Based on the high abundance of *Hc-daf-5* in L3s and its functions in xL3 development, *Hc*-DAF-5 localization in L3s was attempted but failed in this study. Therefore, protein localization was performed on adult worms. Herein, native *Hc*-DAF-5 protein was expressed prominently in the reproductive organs, which is consistent with *Hc*-DAF-3 [38]. Considering that *Ce*-DAF-5 is required for egg-laying [5], it was proposed that *Hc*-DAF-5 may also function in embryonic development and spermatogenesis. In addition to the gonad organs, *Hc*-DAF-5 was also expressed in the cuticle and intestine of adult worms. In *H. contortus*, the cuticle enables the worms to be resistant to harmful substances [40]. In summary, *Hc*-DAF-5 may also be associated with such processes as embryonic development and environmental resistance.

Phylogenetic analysis in this study revealed a relatively close relationship between DAF-5s in the free-living nematodes and parasitic species. In *C. elegans*, DAF-5 functions as a co-factor of DAF-3 (co-Smad), rather than an antagonist. However, its homologue functions as an antagonist of co-Smad in vertebrates [3]. The relatively close relationship between *Hc*-DAF-5 and *Ce*-DAF-5 suggested that *Hc*-DAF-5 probably also functions as a co-factor of *Hc*-DAF-3. In order to test this hypothesis, BiFc was used for verifying the interaction between *Hc*-DAF-5 and *Hc*-DAF-3. The results showed that the region downstream of the SDS box of *Hc*-DAF-5 was dispensable for *Hc*-DAF-3 binding, and the MH2 domain of *Hc*-DAF-3 was sufficient for its interaction with *Hc*-DAF-5, consistent with the findings in *C. elegans* and vertebrates [5], suggesting that *Hc*-DAF-3 and *Hc*-DAF-5 interact with each other and function as a transcriptional regulatory complex to further regulate the downstream transcription.

## Conclusions

In conclusion, a transcription factor *Hc-daf-5* in *H. contortus* was investigated. This gene was transcribed throughout the life cycle of this parasitic nematode, with the highest level in L3s, and the native *Hc*-DAF-5 was expressed in the reproductive organs, cuticle, and intestine of adult worms. In addition, knockdown of *Hc-daf-5* retarded larval development in vitro. Moreover, the interaction between *Hc*-DAF-5 and *Hc*-DAF-3 was also verified. In conclusion, these results provide evidence that *Hc-daf-5* plays an important role in the development of *H. contortus*.

## Supplementary Information

**Additional file 1: Table S1.** PCR Primers used in the present study. **Table S2.** Sequences of DAF-5 homologues used for alignment and phylogenetic analyses. **Table S3.** Sequences of *Hc-daf-5*-specific siRNA and control siRNA used in RNAi.

## Data Availability

The data supporting the conclusions of this article are provided within the article.
