# Supplementary material for: A transcription factor DAF-5 functions in Haemonchus contortus development
Source: Parasit Vectors. 2021 Oct 12;14:529. doi: 10.1186/s13071-021-05036-2 (PMC8507387; doi:10.1186/s13071-021-05036-2)
Supplement: Supplementary file 1 — Additional file 1: Table S1. PCR Primers used in the present study. Table S2. Sequences of DAF-5 homologues used for alignment and phylogenetic analyses. Table S3. Sequences of Hc-daf-5-specific siRNA and control siRNA used in RNAi. [file 13071_2021_5036_MOESM1_ESM.docx]

**Table S1**

Oligonucleotide primers (5’-3’) used in the present study

| Primer | Sequence (5’-3’) |
| --- | --- |
| For *Hc-daf-5* gene isolating | |
| Hc-daf-5-cF | ATGGCCGCTGCCACACAGGATGCT |
| Hc-daf-5-cR | TTATTGCGTTAGTTGTGGTGCAG |
| For transcriptional level analysis | |
| Hc-daf-5-qF | AAGCGCTAGAATCCGTCCTG |
| Hc-daf-5-qR | CAATCGCTCGTACTCTGCCA |
| Hc-tubulin-qF | TGTTCCATCACCCAAGGTATCC |
| Hc-tubulin-qR | TGACAGACACAAGGTGGTTGAGAT |
| For prokaryotic expression | |
| Hc-daf-5-pF | GAATTCGATGCGGAAGTATCAGGCACTC |
| Hc-daf-5-pR | CTCGAGATCCGAGAAGATAGGTAATGGCT |
| For RNA interference in *H. contortus* | |
| Hc-18S-qF | AATGGTTAAGAGGGACAATTCG |
| Hc-18S-qR | CTTGGCAAATGCTTTCGC |
| For BiFc | |
| Hc-daf-3-fF | TGAACCGTCAGATCCGCTAGCCACCATGCGAAGCTCATCCGCT |
| Hc-daf-3-fR | ACATCGTAAGGATATCTCGAGCCTATTTCATGCATCAGTTCGTC |
| Hc-daf-5-fF | TGAACCGTCAGATCCGCTAGCCACCATGGCCGCTGCCACACAG |
| Hc-daf-5-fR | TCAGCTTCTGCTCGCCGATCGCTTGCGTTAGTTGTGGTGCAGA |
| Hc-daf-3-MH2-F | TGAACCGTCAGATCCGCTAGCCACCATGTCAAATTGGGGATGCTT |
| Hc-daf-5-SDS-R | TCAGCTTCTGCTCGCCGATCGCAGTAGACGGATCCGAGAA |

Underscore represents restriction sites.

**Table S2**

Sequences of DAF-5 homology used for alignment and phylogenetic analysis

| Species | GenBank accession number | Reference |
| --- | --- | --- |
| *Ancylostoma ceylanicum* | SKI EYB97253.1 | [26] |
| *Brugia malayi* | Snowski AAQ93809.1 | [5] |
| *Caenorhabditis briggsae* | DAF-5 XP_002631643.1^1^ | [27] |
| *Caenorhabditis elegans* | DAF-5 NP_496941.1^1^ | [28] |
|  | DAC-1 NP_001021129.1^1^ | [29] |
|  | MAB-31 NP_491129.1^2^ | [29] |
| *Danio rerio* | SKI NP_571013.1^1^ | [30] |
|  | Dachshund XP_009304051.1^1^ | [31] |
| *Drosophila melanogaster* | Snowski ABV53643.1^1^ | [11] |
|  | Iceskate NP_651946.1^1^* | [32] |
|  | Dachshund NP_723968.1^1^ | [33] |
| *Equus caballus* | SKI NP_001075287.1 | [34] |
| *Homo sapiens* | SKI NP_003027.1^1^ | [35] |
|  | Icy XP_292349.1^1^* | Unpublished |
|  | Skate XP_064560.4^1^* | Unpublished |
|  | SnoN NP_005405.2^1^ | [10] |
|  | DACH-1 NP_542937.3^1^ | [36] |
| *Mus musculus* | SKI NP_035515.2^1^ | [37] |
|  | Dachshund NP_001033699.1^1^ | [38] |
| *Nippostrongylus brasiliensis* | SKI NBR_0001757401^1^  (found in Wormbase) | [39] |
| *Toxocara canis* | SKI KHN86433.1^1^ | [40] |

^1^ Sequence was used for phylogenetic analysis.

^2^ Sequence was used as an outgroup for phylogenetic analysis.

* The genomic sequence of this record was removed because it has been superseded by a new assembly of the genome, but the GenBank accession of the new sequence is not available now.

**Table S3** Sequences of *Hc-daf-5*-specific siRNA and control siRNA

| Name | Sequence | Targeted regions |
| --- | --- | --- |
| S1 siRNA | Sence: 5’- GGAAGUAUCAGGCACUCAATT -3’ | 327-345 bp |
|  | anti-sense: 5’-UUGAGUGCCUGAUACUUCCTT -3’ |  |
| S2 siRNA | Sence: 5’-GCUGGUGGGCAACUUAUUUTT -3’ | 1045-1063 bp |
|  | anti-sense: 5’-AAAUAAGUUGCCCACCAGCTT -3’ |  |
| S3 siRNA | Sence: 5’-GCACAACAGCUAAUGCAAUTT-3’ | 1582-1600 bp |
|  | anti-sence: 5’-AUUGCAUUAGCUGUUGUGCTT-3’ |  |
| Control siRNA | Sence: 5’-UUCUCCGAACGUGUCACGUTT -3’ | Not applicable |
|  | anti-sence: 5’-ACGUGACACGUUCGGAGAATT -3’ |  |
